# PEDOT:PSS polymer functionalized carbon nanotubes integrated with graphene oxide and titanium dioxide counter electrode for dye-sensitized solar cells

**DOI:** 10.1016/j.heliyon.2025.e42272

**Published:** 2025-01-25

**Authors:** A M Mahmudul Hasan, Md. Abu Bin Hasan Susan

**Affiliations:** a Department of Chemistry, University of Dhaka, Bangladesh; b Dhaka University Nanotechnology Centre (DUNC), University of Dhaka, Bangladesh

**Keywords:** Dye-sensitized solar cell, Counter electrode, Quaternary composite, Carbon nanotube, Graphene, Conducting polymer

## Abstract

This study aims at developing platinum-free dye-sensitized solar cells (DSSCs). A novel quaternary composite comprising conductive Poly(3,4-ethylenedioxythiophene): polystyrene sulfonate (PEDOT:PSS) functionalized multiwalled carbon nanotube (f-MWCNT), nitrogen-doped reduced graphene oxide (Nr-GO), and titanium dioxide (TiO_2_) has been presented as a cost-efficient counter electrode (CE) of DSSC. The conductivity and structural property of MWCNT have been tailored by blending it with PEDOT:PSS. A small (25 wt%) incorporation of conductive polymer reduce agglomeration and increase the solution dispersity of MWCNTs that hinders due to the van der Waals and π-π stacking interaction of the individual tube. The reduced agglomeration facilitates efficient electron transport pathways as characterized by the electrochemical impedance spectroscopy (EIS) and electron imaging techniques. Among the 3 studied quaternary composites the 2:1:1 ratio of f-MWCNT/Nr-GO/TiO_2_ (composite-1) shows a superior performance with a charge transfer resistance (*R*_CT_) of 7.27 Ω cm^2^ and a cathodic peak current density (*J*_PC_) of −17.08 mA cm^−2^. The cell achieves a noteworthy photoconversion efficiency (PCE) of 4.25 ± 0.32 % under standard test conditions at AM1.5G. The PCE of this quaternary composite is comparable to Pt thin film (5.53 ± 0.24 %) and 9 % higher than f-MWCNT/Nr-GO binary composite. The DSSC, constructed using this quaternary composite CE, displays extended cyclability and a prolonged chemical stability even after 500 reversible redox cycles.

## Graphical abstract

**Figure:**
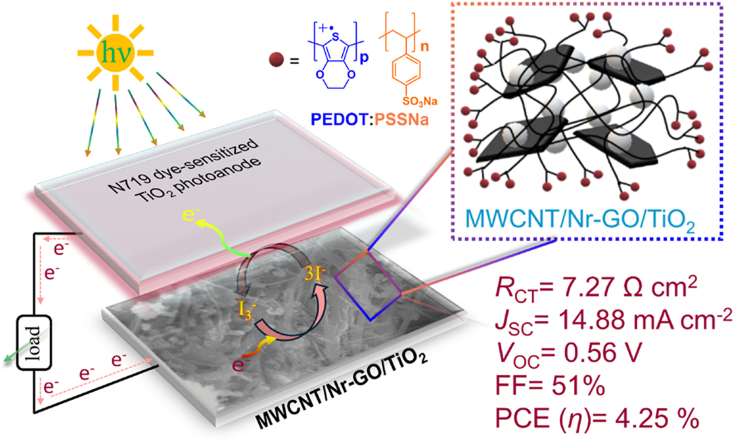

## 1 Introduction

The urgent necessity to shift from traditional energy systems reliant on fossil fuels to cleaner alternatives has brought innovation in renewable technology. Advances in renewable photovoltaics have offered rise to a promising substitute in the form of dye-sensitized solar cells (DSSCs), which provides a distinctive combination of efficiency, cost-effectiveness, and environmental sustainability [[1], [2], [3], [4]]. Moreover, the straightforward working principle makes them more appealing compared to other renewable photovoltaics i.e., perovskite, organic and Si- based solar cells. Unlike perovskite cells, which often include lead, DSSCs use largely non-toxic elements like titanium dioxide (TiO_2_), and carbon materials reducing their environmental impact. Although DSSCs are often less efficient than silicon and perovskite cells, they perform exceptionally well in low light conditions, which makes them appropriate for indoor or diffused light applications and wearable electronics [[5], [6], [7], [8], [9], [10]].

In DSSC the photoanode typically made of TiO_2_, contains organic metal complex dyes (i.e., N719, N3, C101, [Ru(bpy)_2_(phen)]^2+^, [Co(bipy)_3_]^2+^) that are crucial for photon absorption [[11], [12], [13]]. Besides synthetic metal complexes, natural organic dyes like chlorophylls, carotene, and lutein have been explored in various studies [14,15]. However, these natural dyes tend to degrade over time, which can negatively impact the efficiency of the cells. The photogenerated electrons upon photon absorption jump from the highest occupied molecular orbital (HOMO) of the sensitized dye (i.e., N719) to the conduction band (CB) of the photoanode, then travel through the outer circuit to the counter electrode (CE). The counter electrode (CE), composed of conductive materials like platinum (Pt), completes the circuit by receiving the photogenerated electrons and assist reduction of the oxidized ${\;I}_{3}^{-}$ to 3 $I^{-}$. The $I_{3}^{-}$/ $I^{-}$ redox mediator restore the dye by donating electrons during oxidation, reverting the photo-oxidized dye to its original condition, so enabling it to absorb further light and perpetuate the cycle [[16], [17], [18], [19], [20]].

The photoconversion efficiency of DSSC significantly depends on the $I_{3}^{-}$ reduction kinetics and I_2_ adsorption feasibility on the CE surface [[21], [22], [23]]. Materials with superior conductivity and surface area facilitate the $I_{3}^{-}$ reduction with minimal charge recombination. While platinum is commonly used because it meets these requirements, its high expense has driven researchers to explore affordable carbon-based CEs as alternatives. Carbon materials are abundant in nature, easy to process and demonstrate a high degree of electrochemical stability [[24], [25], [26]]. Recently, significant attention has been given to find Pt-free alternative electrocatalytic materials including carbonaceous materials (e.g., carbon nanotube, graphene, carbon dot, activated carbon) [[27], [28], [29], [30], [31]], transition metal compounds (e.g., carbides, nitrides, chalcogenides, oxides) [32], and conducting polymers (e.g., PEDOT, polyaniline, polypyrrole) [33]. In addition, composite counter electrodes have attracted interest due to the synergistic effects of each component, which enhance electron transport and accelerate the I₃⁻ reduction kinetics [[34], [35], [36], [37]].

Carbon nanotubes (CNTs) offer faster carrier movement due to the delocalized π electrons of sp^2^ hybridized carbon, increased surface area from their hollow structure, and notable chemical inertness [38]. However, their tendency to aggregate and poor solubility pose challenges for their practical application in DSSCs [39]. This work involves functionalization of MWCNTs with poly(3,4-ethylenedioxythiophene) (PEDOT):poly(styrene sulfonate) (PSS), forming a conductive ionic polymeric network of PEDOT:PSS. The ionic interaction resulting from the negatively charged sulfonate group within this conductive polymer allows for better dispersion in typical polar organic solvents (e.g., ethanol, N-methyl-2-pyrrolidone, dimethylacetamide, dimethylformamide) and provides structural integrity for composite formation. Xiao *et* al. [40] observed a 20 % increase in cell performance when MWCNTs were blended with conductive PEDOT polymer. This enhancement stems from the improved intrinsic electrical conductivity and ease of processing of the electrode materials. Yun *et* al. [22] have demonstrated that the inclusion of MWCNT in PEDOT:PSS leads to a substantial enhancement in both electronic and ionic conductivity. The elevated surface roughness enhances the electrocatalytic activity of the composite towards $I_{3}^{-}$ reduction and gain a cell efficiency of 7.29 %.

Graphene, another carbon material, shows promising electrical conductivity and surface properties [41]. Xue *et* al. [42] demonstrated how the two-dimensional surface of nitrogen-doped graphene facilitates electron polarization at the solid electrode-electrolyte interface, favoring the reduction of $I_{3}^{-}$ at low overpotential elevating the cell performance by 70 %. Semiconductor oxides show potential as photoanode materials due to their tunable bandgap, enabling effective photon absorption [43,44]. They can also serve as CEs in composite materials, providing structural stability and increasing overall surface area. The crystalline structure of the semiconductor oxide provides higher $I_{3}^{-}$ adsorption site, resulting in a decrease in the Gibbs free energy of adsorption, Δ*G*_ads_ and favoring the reduction of $I_{3}^{-}$. Quantum calculation shows that the adsorption energy of iodine on α-Fe_2_O_3_ is about the same as that on a Pt counter electrode at the acetonitrile/electrode interface [35]. Chew *et* al. [45] identified MWCNT/ZnO as a promising CE with a PCE ranging from 0.46 % to 7.07 %. Similarly, some recent studies have demonstrated that the CE designs incorporating conductive polymers with conductive carbon or semiconducting metal oxide materials significantly enhance the cell performance [[46], [47], [48], [49]]. Our previous work [50] highlighted how modifying the surface properties of MWCNTs with carbon dots significantly enhance cell efficiency. This work builds upon our previous findings, representing a continued effort to advance DSSC technology.

**Scheme 1:**
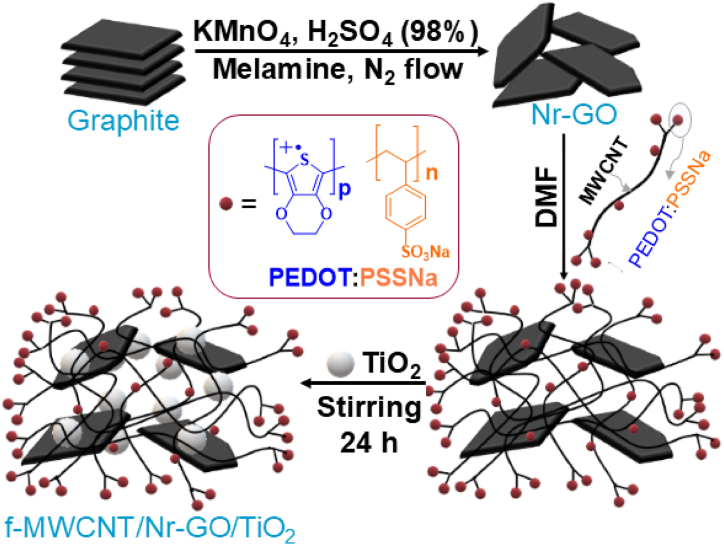
Schematic diagram for synthesis of f-MWCNT/Nr-GO/TiO_2_ quaternary composite. (The black line represents the MWCNT, while the red dot signifies the PEDOT:PSS polymer, which is randomly distributed over the tube).

In this work, we have synthesized a quaternary composite consisting of PEDOT:PSS polymer-functionalized MWCNT (f-MWCNT), N-doped reduced graphene oxide (Nr-GO), and TiO_2_ nanorods (NR) (f-MWCNT/Nr-GO/TiO_2_) at various component ratios for use as a CE in DSSCs (as depicted in Scheme 1). In our electrode design, we prioritized the significant surface area and conductivity of carbon-based materials, along with the mixed ionic-electronic conduction properties of PEDOT:PSS [22]. A semiconducting oxide was integrated into the polymeric network to establish nanoscale electronic conduction pathways, enhancing charge delocalization within the nanostructure and accelerating the reduction kinetics of $I_{3}^{-}$ [51]. In this research, MWCNTs were functionalized with 25 wt% of PEDOT:PSS to enhance their ionic conductivity and solution processability. The percent incorporation of PEDOT:PSS into MWCNT has been validated by the ionic conductivity study. Combining materials in composites often leads to improved electrical conductivity, increased surface area, and greater stability compared to binary or ternary counterparts. Additionally, incorporating multiple components allows for greater customization of electrode properties to enhance performance metrics such as fill factor and PCE. The quaternary system was developed to create an electrode with improved charge transfer efficiency, greater stability, and enhanced structural flexibility compared to binary and ternary hybrids. Jennifer *et* al. [51] proposed a quaternary composite counter electrode comprising r-GO/MnO₂/NiO/CuO, which demonstrated a PCE reaching 93.31 % of Pt-CE under same conditions and higher than the binary and ternary composites.

In this study, the electrocatalytic performance of each component of the quaternary composite has been vastly studied through electrochemical impedance spectroscopy (EIS), cyclic voltammetry (CV) and the current voltage (*J-V*) behavior analysis of the cell. The studied quaternary composite is unique from the design of sprinkling crystalline semiconductor nanoparticles over the 3D network of conducting polymer functionalized carbon nanotube and graphene nanosheet. The addition of TiO_2_ to the composite improves its structural integrity and increases the number of available I_2_ adsorption sites. This, in turn, raises the PCE of the cell, as evidenced by the *J-V* profile analysis.

## 2 Experimental

### 2.1 Materials

Multi-walled carbon nanotube (length: 5 μm; diameter: 6–9 nm), poly(3,4-ethylenedioxythiophene):polystyrene sulfonate, N, N-dimethylformamide (DMF) were purchased from Sigma Aldrich for functionalization of MWCNT. Graphene oxide (GO) was synthesized using graphite powder (commercial grade), and the other chemicals were received as follows: NaNO_3_ (Sigma Aldrich), H_2_SO_4_ (98 % solution; Sigma Aldrich), KMnO_4_ (commercial grade), H_2_O_2_ (30 % solution; Sigma Aldrich), and deionized water (HPLC grade; at 25.0 °C conductivity of 0.055 μScm^−1^) from BOECO (Germany) water purification system. Graphene oxide was reduced to N-doped reduced graphene oxide (Nr-GO) using melamine powder (Sigma Aldrich), HCl (37 % solution; Alfa Aesar) and formaldehyde solution (Sigma Aldrich). TiO_2_ rutile powder (Fluka, Research Chemicals) and NaOH flakes (research-grade) were used to synthesize TiO_2_ nanorod (NR). Functionalized MWCNT, synthesized N-doped reduced graphene oxide, and TiO_2_ NR were used for the synthesis of f-MWCNT/Nr-GO/TiO_2_ NR quaternary composite.

### 2.2 Functionalization of MWCNTs

The MWCNTs were ultrasonicated for 30 min in dry DMF to have a dispersion at a concentration of 3 % (w/w) in the form of a black slurry. A PEDOT:PSS solution in DMF at a concentration of 9 % (w/w) was then gradually added to the black slurry at a weight ratio of 25 %. The dispersion was magnetically stirred for 30 min followed by sonication for 4 h to form a dark paste-like substance. The sample was then dried for 24 h at 110 °C to remove any excess DMF (boiling point = 154 °C). In the dried sample, the proportions of MWCNTs and PEDOT:PSS were approximately 75 % (w/w) and 25 % (w/w), respectively.

### 2.3 Synthesis of graphene oxide

The modified Hummer process was used to prepare GO from graphite powder [52]. Graphite powder (1.50 g) and NaNO_3_ (0.75g) were mixed in a flask, and 50 mL H_2_SO_4_ (95 %) was added to it, which was subsequently placed in an ice-bath and agitated. To prevent overheating, 4 g of KMnO_4_ were intermittently added to the suspension while agitating at ambient temperature for 2 h. Upon the suspension turned to a vibrant shade of brown color, a gradual addition of 45 mL of distilled water was made to avoid bumping. Once the suspension attained a temperature of approximately 120 °C, the color changed to yellow. The resultant suspension was magnetically stirred for 15 h at 140 °C. 30 % H_2_O_2_ (15 mL) was then added to the mixture. Rinsing with deionized water and a 5 % HCl solution on multiple occasions facilitated the purification of GO and a black powder was obtained after filtration and drying.

### 2.4 Reduction of GO to Nr-GO

0.5 g of GO powder was dispersed into 50 mL water to reduce it into Nr-GO. After charging the resulting dispersion into a second flask, 15 g of melamine powder and 30 mL of formaldehyde solution were added to the mixture with constant stirring. The mixture, upon heating at 100 °C, became clear. After stirring for 1 h, the mixture was cooled down to 40 °C and HCl solution was added to bring the pH level of the mixture down to 4.5. The mixture was kept for 16 h in a static condition for the formation of GO-melamine composite. The final product was filtered and rinsed with ethanol and water. The product was dried for 24 h in an oven at 60 °C. After cooling down to 40 °C, HCl solution was added to adjust the pH level of the mixture. The finished powdered product was heated for 2 h at 5 °C/min and N_2_ flow rate of 200 mL/min in a tube furnace to obtain the N-enriched reduced GO. A thermogravimetric measurement of the synthesized GO-melamine composite was conducted to evaluate the decomposition temperature of GO. The final obtained sample was free of melamine and was stored for characterization.

### 2.5 Synthesis of TiO_2_ nanorod

TiO_2_ nanorod was synthesized by sol-gel method. 2 g TiO_2_ was dispersed in 50 mg/mL NaOH solution, and vigorously stirred for 24 h. The resultant suspension was placed into an alumina crucible and held in a static position for the setting of sol-gel. It was then dried at 60 °C and calcined in a muffle furnace for 2h at 600 °C (heating rate: 5 °C/min).

### 2.6 Synthesis of f-MWCNT/Nr-GO/TiO_2_ composite

The f-MWCNT, synthesized Nr-GO, and TiO_2_ were evenly dispersed in 50 mL DMF through sonication for 2 h. The resulting suspension upon continuous and vigorous stirring for 24 h turned into a gray color paste. This was heated at 110 °C in an oven for 24 h to remove excess DMF. Thus, three quaternary composites of f-MWCNT/Nr-GO/TiO_2_ were prepared by varying the amount of the individual component. These are: (a) composite-1 (f-MWCNT/Nr-GO/TiO_2_ = 2:1:1) (b) composite-2 (f-MWCNT/Nr-GO/TiO_2_ = 1:2:1) and (c) composite-3 (f-MWCNT/Nr-GO/TiO_2_ = 1:1:1). The synthesis of the composite has been schematically shown in Scheme 1. A binary composite consisting of f-MWCNT and Nr-GO was also synthesized following a similar procedure, with a weight ratio of 2:1. This composite is denoted as f-MWCNT/Nr-GO-21.

### 2.7 Electrochemical measurements

The electrochemical investigation was performed using a three-electrode cell. A 2 mm Pt disk WE, Pt wire CE and Ag/Ag^+^ nonaqueous reference electrode were used. 0.5 M LiI, 0.05 M I_2_, and 0.1 M LiClO_4_ were dissolved together in acetonitrile to prepare electrolyte solution, which was purged with continuous N_2_ flow during CV and EIS experiments. The composite material (95 wt %) was mixed with polyvinylidene fluoride (PVDF; 5 wt %) binder in 100 μL ethanol and sonicated for 30 min. The mixture was drop-casted onto the polished Pt disk electrode surface. The electrode was allowed to dry under ambient condition for 24 h followed by heating at 80 °C for 2 h. The EIS and CV measurements were conducted using an electrochemical analyzer (CHI 760E; CH Instruments, USA). A Pt disk WE was used for each measurement, with a consistent sample size of 2.0 mg. EIS measurements were carried out between 1 Hz and 1 MHz.

### 2.8 Preparation of dye coated TiO_2_ photoanode

A thin layer of TiO_2_ was coated on fluorine doped tin oxide (FTO). A clean FTO substrate was immersed in a 40 mM aqueous titanium butoxide Ti(OBu)_4_ solution for 30 min at 80 °C. 10 mL of ethanol was used for dispersing 0.5 g of TiO_2_ powder and 5 wt% of perfluorinated resin (nafion) binder. The resultant paste was applied to the FTO using the doctor blade technique (over the blocking layer; surface area 1 cm^2^; thickness 6–9 μm). The substrate was dried at 120 °C for 15 min followed by annealing for 15 min at 450 °C. The produced photoanode was immersed for 16 h in a 0.5 mM N719 dye solution in acetonitrile and the electrode was washed with deionized water and dried for 5 min under nitrogen. (Fig. S12, see supporting information for more details). We used the N719 inorganic metal complex dye because of its prolonged chemical stability and wide range of photon absorption (300–700 nm) in the visible spectrum (Fig. S1).

### 2.9 Preparation of the composite CE

The f-MWCNT/Nr-GO/TiO_2_ quaternary composite (20 mg) was mixed with 5 wt% nafion binder in 4 mL ethanol and stirred for 30 min and transferred to a mortar to transform into a sticky paste. Using a doctor blade technique, a thick layer (6–9 μm) of the paste was applied to a clean surface of FTO glass substrate. The coating covered an area of 1 cm^2^. The substrate was subsequently dried for 30 min at 80 °C under N_2_.

### 2.10 DSSC assembly

The dye-coated TiO_2_ photoanode and the f-MWCNT/Nr-GO/TiO_2_ fabricated photocathode were securely fastened together. A surlyn spacer was positioned around the active area (0.25 cm^2^). The electrolyte, I⁻/I₃⁻ was introduced into the cell and the photovoltaic performance was evaluated by conducting the current-voltage (*J*-*V*) analysis (ZAHNER, Xpot, 26356; Germany) under irradiation of simulated sunlight (AM 1.5, 100 mW cm⁻^2^) (Fig. S13).

## 3 Results and discussion

### 3.1 Characterization of materials

**Fig. 1:**
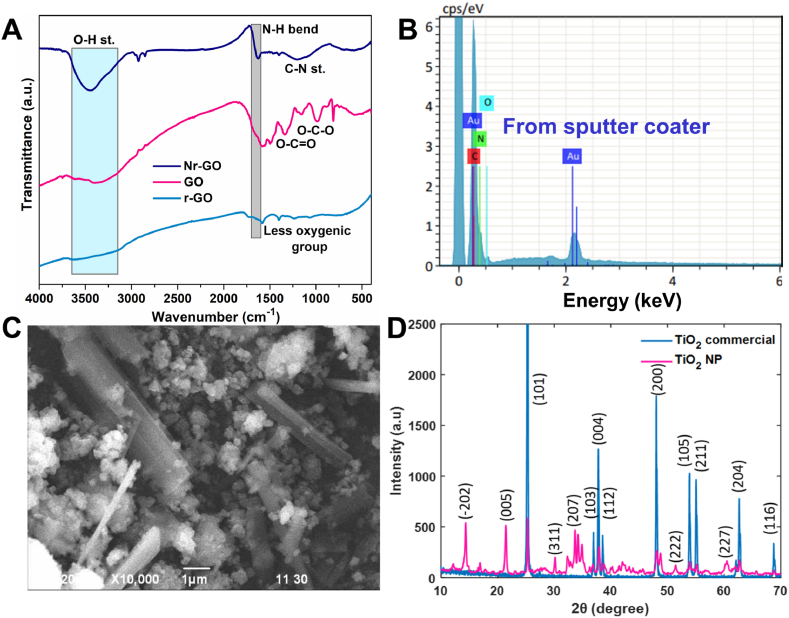
(A) Transmission spectra of GO, r-GO, and Nr-GO and (B) Energy dispersive X-ray spectrum of Nr-GO to confirm successful N doping. Characterization of thermally treated TiO_2_ by (C) SEM and (D) XRD analysis.

Chemical oxidation and external melamine dopant successfully produce Nr-GO from graphite. The FTIR spectrum of GO (Fig. 1A) shows the formation of different oxygenic groups after chemical oxidation of graphite. The vibrational bands in 1734, 1600, 1390, 1225, and 1060 cm^−1^ can be assigned to the stretching of O-H in H_2_O adsorbed in the sample, C=O (carbonyl), C=C (aromatic) in the skeleton of GO, O-C=O (carboxyl), O-C-O (epoxy), and C-O (alkoxy) groups, respectively. For Nr-GO, two new bands at 1630 and 1180 cm^−1^ correspond to the N-H bending and stretching of the C-N bond [53]. The band for the O-H stretching is broadened, due to the overlap with the N-H stretching band at 3475 cm^−1^.

For both r-GO and Nr-GO, the bands of C=O, O-C=O, and O-C-O exhibit a lowering in intensity ascribable to the decreasing content as oxygen atoms are consumed due to the reaction between melamine and oxygen-containing functional groups. Melamine, with an electron-withdrawing aromatic ring, facilitated the reduction of GO to r-GO and Nr-GO. The missing bands in r-GO and Nr-GO confirm the reduction of GO and the appearance of new bands (N-H, C-N) in the Nr-GO confirm the nitrogen doping in the r-GO structure. Fig. 1B also proves successful doping of N in Nr-GO from melamine source at very high temperature. There are 15 % N atoms present in the structure of Nr-GO. Fig. S5 depicts a broad lower intensity peak in the X-ray diffraction (XRD) pattern of GO compared to that of the precursor graphite powder. This broad lower angle shifted peak represents the larger interlayer spacing originated from different oxygen-containing groups intercalating in the GO interlayer. When GO is reduced to Nr-GO, XRD pattern shows an intense peak at 26.78°, which indicates the interlayer spacing of 3.3 Å (calculated by using Bragg's equation: λ = 2dsinθ), identical to that of the graphite [54]. Upon reduction, the XRD pattern demonstrates a shift towards higher diffraction angles, indicating reduction of oxygen containing groups in the Nr-GO layer and the subsequent closer proximity of the layers.

Scanning electron microscope (SEM) image of thermally treated TiO_2_ shows the formation of nanoparticles and nanorod (Fig. 1C) during sol-gel and thermal treatment. The formation of the nanorod is also further confirmed by the XRD analysis. Diffraction patterns of both commercial TiO_2_ and synthesized TiO_2_ NR were compared regarding the JCPDS card no. 21–1272 and 76–1648 database. The XRD pattern of calcined TiO_2_ in NaOH (Fig. 1D) shows new peaks at 14, 22 and 38° corresponding to the newly generated sodium titanate (Na_2_Ti_7_O_15_) nanorod [55]. The dynamic light scattering (DLS) profile of TiO_2_ (Fig. S3) shows that the nucleation of nanoparticles increases with temperature and forms the nanorod. This can also be validated by the SEM image of TiO_2_ (Fig. S4). Noticeably, at 600 °C the particle size is smaller than 400 °C. It indicates that at higher calcination temperature, TiO_2_ form nanoparticles and the nanoparticles transform into nanorod through nucleation process [56].

**Fig. 2:**
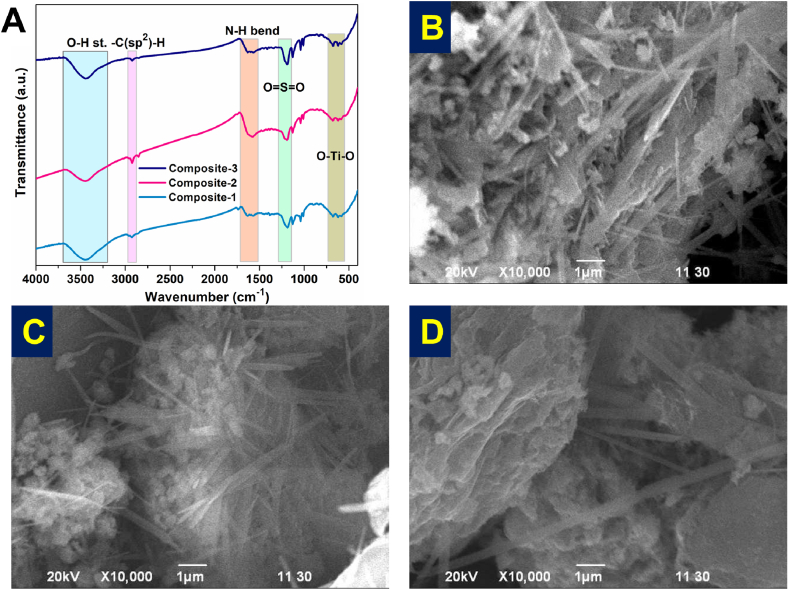
(A) Transmission spectra of three different f-MWCNT/Nr-GO/TiO_2_ composites. SEM image of (B) composite-1, (C) composite-2, and (D) composite-3.

Three quaternary composites of f-MWCNT/Nr-GO/TiO_2_ at different stoichiometry ratio of the individual components were synthesized via sonochemical method. The synthesized composites were examined by spectroscopic and electron imaging techniques. The FTIR spectra of the composites (Fig. 2A) are similar. The band at 3450 cm^−1^ is attributed to the O-H stretching and that of 2920 and 2852 cm^−1^ to symmetric and asymmetric stretching of C-H, respectively. The band at 1600 cm^−1^ corresponds to N-H bending, IR active phonon mode of MWCNT and 1190-1014 cm^−1^ to O=S=O stretching and 640 cm^−1^ to the bending of Ti-O-Ti. C-N bending and O=S=O stretching bands overlapped within the range of 1240-1160 cm^−1^. The composite sample contains the absorption bands of f-MWCNT, Nr-GO, and TiO_2_ sample. It gives an interpretation of the significant incorporation of TiO_2_ NR in the 3D network structure of the carbonaceous composite materials while maintaining the integrity of their backbone structure with exceptional chemical inertness and thermal stability (Fig. S8).

Fig. 2B–D depict the distribution of TiO_2_ nanorod over the 3D network of MWCNT and Nr-GO in the quaternary f-MWCNT/Nr-GO/TiO_2_ composites. The TiO_2_ nanorods are dispersed, interspersed throughout a compact block formed by the f-MWCNT/Nr-GO matrix. This suggests a complex interplay between the individual components, leading to the formation of distinct regions within the composite. Importantly, the presence of TiO_2_ nanorods within the compact block creates a rigid charge transfer channel, indicating the potential for efficient electron transport within the material. Therefore, the resulting nanocomposites have both the defect-rich surface for enhanced catalytic activity and an interconnected network morphology for being an excellent electrical conductor.

**Fig. 3:**
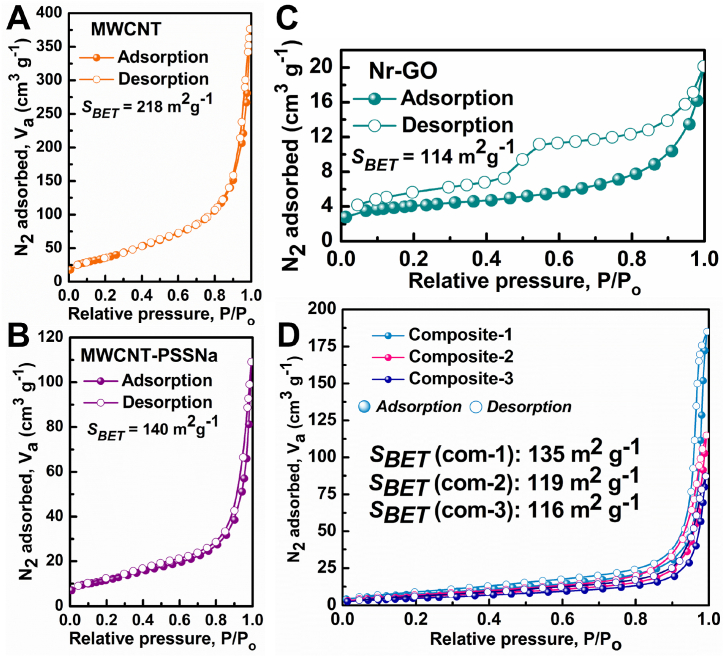
N_2_ sorption isotherm of (A) pristine MWCNT (B) MWCNT-PSS (C) Nr-GO and (D) three different quaternary composites.

The surface properties of the f-MWCNT/Nr-GO/TiO_2_ composites were characterized by the nitrogen adsorption–desorption isotherms analysis following the Brunauer–Emmett–Teller (BET) method. The associated pore size distribution is shown in Fig. S6. The synthesized material demonstrates microporosity, with its surface area significantly influenced by the component ratio within the quaternary composite. The adsorption isotherm of MWCNT aligns with the type III classification according to the 1985 IUPAC criteria [57] (Fig. 3A), indicating a macro-porous structure (*S*_BET_ = 218 m^2^g^-1^) capable of adsorbing a large volume of N_2_ gas. Conversely, the adsorption isotherm of MWCNT-PSS (Fig. 3B) also resembles type III, suggesting a nanoporous surface that adsorbs a lesser quantity of N_2_ gas. The incorporation of PEDOT:PSS polymer reduces surface area of MWCNT from 218 to 140 m^2^g^-1^ and pore volume from 0.58 to 0.16 cm^3^g^-1^, as evidenced by Fig. S6. This reduction is attributed to the obstruction of the porous structure of MWCNT by the PEDOT:PSS polymer. However, the introduction of this conducting polymer enhances the electrical conductivity of the composite, thereby facilitating charge transfer kinetics in the CE of the DSSC [[58], [59], [60]]. The adsorption isotherm of Nr-GO (Fig. 3C) resembles type IV isotherm indicating a mesoporous structure with nominal porosity and pore volume in accordance with of the IUPAC classification (Fig. S6) conducive to capillary condensation. The sorption isotherms of all three quaternary nanocomposites (Fig. 3D) display characteristics akin to both type I and IV isotherms suggesting the formation of a microporous to mesoporous structure with pore volume ranging from 0.10 to 0.30 cm^3^g^-1^ (Fig. S7). The amalgamation of micro-porous f-MWCNT, mesoporous Nr-GO, and TiO_2_ yields a resultant composite with a mesoporous structure, facilitating enhanced contact sites for electrolyte solution adsorption, particularly beneficial for I_2_ adsorption as a CE in DSSCs.

### 3.2 Electrochemical and photovoltaic characterization

The electrocatalytic activity (ECA) of f-MWCNT/Nr-GO/TiO_2_ quaternary nanocomposites toward the I_2_ reduction were studied by the CV and EIS analysis in a three-electrode cell configuration (Fig. S9). The assessment of the ECA of a CE can be made by: cathodic current density (*J*_PC_), cathodic peak potential (*E*_PC_), and the potential difference between the reduction and oxidation process of the $I_{3}^{-}$/ $I^{-}$ redox couple (|*E*_PP_ = *E*_oxidation_ – *E*_reduction_|). Fast kinetics at low overpotential and generation of a substantial amount of cathodic current are indicative of an efficient electrocatalytic reaction. In this regard, ECA shows an inverse correlation with the value of the *E*_PP_ and a direct correlation with the *J*_PC_. A smaller *E*_PP_ corresponds to an enhanced rate constant of a redox process. A higher rate constant signifies a slower charge recombination and a faster charge transfer rate across the electrode/electrolyte interface.

**Fig. 4:**
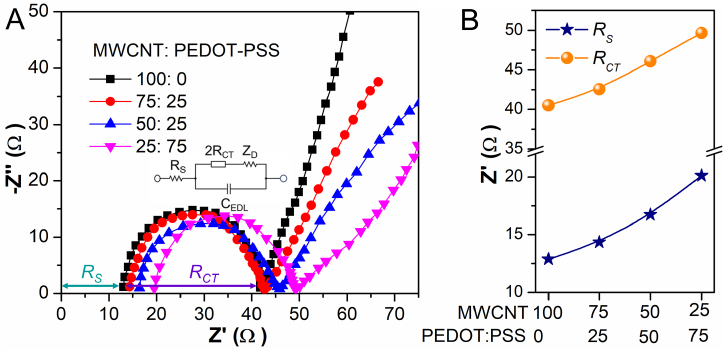
Electrochemical impedance analysis by the (A) Nyquist plot of MWCNT/PEDOT:PSS at different weight % and (B) Extracted *R*_S_ and *R*_CT_ from the Nyquist plot.

The electrochemical conductivity of MWCNT/PEDOT:PSS (f-MWCNT) was assessed by impedance analysis at various weight ratios to determine the impact of using the PEDOT:PSS conducting polymer to functionalize the MWCNT. The EIS was recorded using a symmetric cell configuration made by sandwiching 0.5 M LiI, 0.05 M I_2_, and 0.1 M LiClO_4_ in acetonitrile electrolyte between a MWCNT/PEDOT:PSS cast FTO electrode. The EIS was conducted at a bias voltage of −0.05V and an applied alternating current (AC) frequency ranging from 1 Hz to 100 kHz. The Nyquist plot (Fig. 4A) provides information on the series resistance (*R*_S_) and the charge transfer resistance (*R*_CT_) of the material. These parameters can be utilized to assess the electrocatalytic activity and ionic conductivity of the material. The series resistance is contingent upon the conductivity of the bulk material. Fig. 4 illustrates a decrease in both the *R*_S_ and *R*_CT_ as the MWCNT content increases in the MWCNT/PEDOT:PSS composite. It is hypothesized that an increased concentration of PEDOT:PSS obstructs the active surface area of MWCNT, hence impeding the reduction of $I_{3}^{-}$ at the electrode/electrolyte interface, as illustrated in Fig. 4B. A 75:25 (w/w) MWCNT/PEDOT:PSS system was chosen to functionalize the MWCNT based on the obtained result as this system can enhance the dispersity of MWCNT in polar organic solvents while causing only a slight decrease in the electrocatalytic performance of the material.

**Fig. 5:**
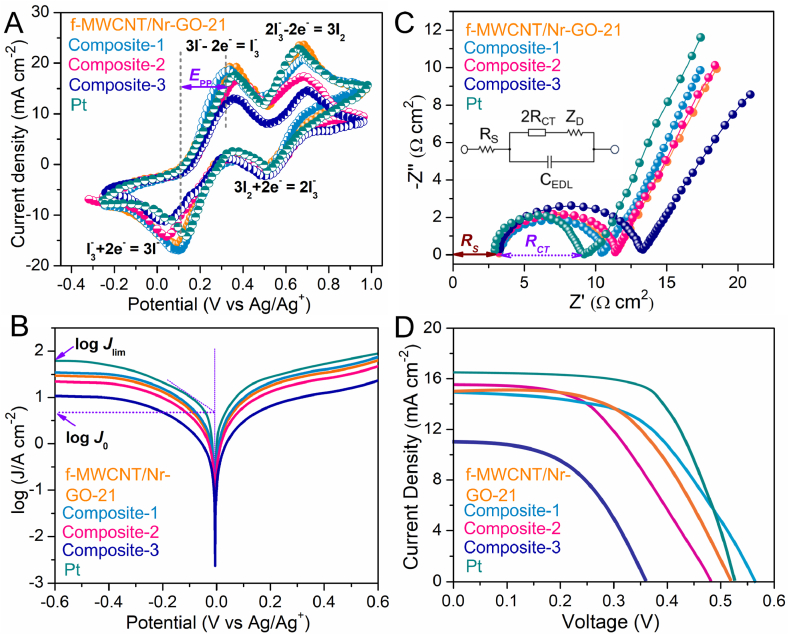
(A) Cyclic voltammogram, (B) Tafel polarization curve, (C) Nyquist plot, and (D) *J-V* analysis curve of three different composites. [orange = f-MWCNT/Nr-GO-21, cyan = composite-1, pink = composite-2 and royal = composite-3, pine green = Pt] (The electrolyte solution consists of 0.5 M LiI, 0.05 M I_2_, and 0.1 M LiClO_4_ in acetonitrile. CV and EIS have been recorded at 50 mV s^−1^ scan rate and 1–10^6^ Hz frequency respectively).

The electrochemical performance of f-MWCNT/Nr-GO/TiO_2_ quaternary composites was compared with the f-MWCNT/Nr-GO-21, f-MWCNT/TiO_2_ (2:1) and Nr-GO/TiO_2_ (2:1) binary and ternary composites. The cyclic voltammogram of all composites (Fig. 5A and Fig. S10A) show two pairs of redox peaks corresponding to four charge transfer reactions of iodine (inset of Fig. 5A). Among these four-electron process, our point of interest is the reduction of $I_{3}^{-}$ assigned with the second reduction trace that takes place on the CE surface that is influenced by the surface characteristics and morphology of the CE material. In Fig. 5A, composite-1 shows the highest value for *J*_PC_ (−17.08 mA cm^−2^) and the lowest for *E*_PP_ (0.23 V), whereas composite-3 demonstrates the lowest *J*_PC_ (−11.46 mA cm^−2^) and the highest *E*_PP_ (0.33 V). The electroreduction of $I_{3}^{-}$ is faster on the surface of composite-1 electrode compared to the f-MWCNT/Nr-GO-21 and f-MWCNT/TiO_2_ ternary and Nr-GO/TiO_2_ binary composites, resulting in the generation of a maximum amount of cathodic current (Table 1). The composite-1 displays superior electrocatalytic activity compared to the other three composites for $I_{3}^{-}$ reduction. It is also worth noting that the *J*_PC_ values for the ternary and binary composites are comparable but show an enhancement in the quaternary composites, whereas an opposite trend is observed for the *E*_pp_. These findings emphasize the superior performance of quaternary composite-1 over the ternary and binary hybrids.

**Table 1:** Parameters evaluating electrocatalytic performance of the nanocomposite modified CE.

Sample	*J*_PC_ (mA cm^−2^)	*E*_pp_ (V)	*R*_S_ (Ω cm^2^)	*R*_CT_ (Ω cm^2^)	*J*_lim_ (A cm^−2^)	*J*_*0*_ (A cm^−2^)
f-MWCNT/Nr-GO-21	−15.41	0.26	3.13	8.24	1.48	0.44
f-MWCNT/TiO_2_	−15.10	0.24	3.13	9.61	1.47	0.42
Nr-GO/TiO_2_	−15.29	0.34	3.13	12.81	1.38	0.39
Composite-1	−17.08	0.23	3.13	7.27	1.54	0.52
Composite-2	−14.23	0.31	3.13	8.29	1.33	0.35
Composite-3	−11.46	0.33	3.13	10.19	1.03	0.18
Pt thin film	−14.16	0.21	2.81	5.84	1.83	0.67

The charge transfer reaction rate at the CE/electrolyte interface was examined by Tafel polarization curves. This technique generates a polarization profile, illustrating the semilogarithmic relationship of current density and potential (log *J* vs. V). The Tafel plot typically features a distinct region, characterized by a significant gradient which governs ECA of the CE. Fig. 5B shows the polarization profile to determine limiting diffusion current density (*J*_lim_) and exchange current density (*J*_0_). Higher values of *J*_lim_ and *J*_0_ suggest superior electrocatalytic performance of the material. Composite-1 shows the maximum values of *J*_lim_ and *J*_0_ close to Pt, indicative of its superior electrochemical activity.

Information regarding the feasibility of charge transfer for $I_{3}^{-}$ reduction on the CE surface of DSSC is given by EIS. The conventional Nyquist plot (Fig. 5C and Fig. S10B) demonstrates an obvious delineated semicircle with a single secondary curve. The solution resistance (*R*_S_) of the CE has been determined from the real axis (Z″) intercept at a high frequency (about 100 kHz) of the CE. The small semicircle at the low-frequency region dictates the Nernst diffusion impedance (*Z*_D_) of an electrolyte, whereas the first semicircle at an intermediate frequency refers to the charge transfer resistance (*R*_CT_) and the corresponding constant phase element (CPE) of the electrolyte and CE [10,61,62]. This work primarily highlights the *R*_CT_ and *R*_S_ values of the CE and the *Z*_D_ value is insignificant. A material with good ECA for $I_{3}^{-}$ reduction is expected to have a lower *R*_CT_ to indicate ease of electron transfer at the interface of the CE/electrolyte. Pt thin film shows the lowest charge transfer resistance (5.84 Ω cm^2^) indicating its superior electrocatalytic activity. Among the three quaternary composites, composite-1 shows the lowest value of *R*_CT_ (7.27 Ω cm^2^) indicating its potential efficiency as a CE in DSSCs compared to two other quaternary, tertiary and binary composites. Furthermore, the steeper Nyquist slope in the low-frequency region of Fig. 5C for both the Pt thin film and quaternary composite-1 suggests higher charge capacitance, indirectly reflecting a larger active surface area for these materials. In contrast, the smaller Nyquist slope for quaternary composite-3 indicates that the active area is obstructed by the 2D graphene nanosheets, consistent with the SEM images and BET surface area analysis. The active surface areas of the materials were also compared by recording their CVs at various scan rates and fitting the data to the Randles–Sevcik equation under identical solution conditions, where ion concentration and diffusion coefficient remained constant (Fig. S11, see supporting information for more details). Quaternary composite-1 exhibited the highest active surface area, while composite-3 showed the lowest. This result is consistent with the CV and Tafel polarization analysis. The performance metrics of the quaternary composite-1 are comparable to Pt thin film CE and superior to the ternary and binary composites (Table 1).

The photovoltaic performance of the f-MWCNT/Nr-GO/TiO_2_ composites as a CE in DSSCs was analyzed under irradiation of simulated sunlight (AM 1.5, 100 mW cm^−2^). The *J*-*V* curves are depicted in Fig. 5D, and analysis matrices are tabulated in Table 2. FF is crucial in determining the maximum power of a solar cell, along with short circuit current (*I*_SC_) and open circuit voltage (*V*_OC_). Maximum power (*P*_max_ = $I_{\text{mp}}\times V_{\text{mp}}$) from the solar cell is divided by the product of *V*_OC_ and *I*_SC_ to have the FF:

(1) $$\text{FF}=\frac{I_{\text{mp}}\times V_{\text{mp}}}{I_{\text{SC}}\times V_{\text{OC}\;}}$$

**Table 2:** Matrices obtained from the *J*-*V* analysis of the manufactured DSSC with different CEs of f-MWCNT/Nr-GO/TiO_2_ quaternary composites.

Sample	*J*_SC_ (mA cm^−2^)	*V*_OC_ (V)	FF (%)	PCE, *η* (%)
MWCNT/Nr-GO-21	15.03 ± 1.89	0.52 ± 0.08	50 ± 2	3.91 ± 0.20
Composite-1	14.88 ± 1.45	0.56 ± 0.10	51 ± 2	4.25 ± 0.32
Composite-2	15.28 ± 1.83	0.48 ± 0.07	48 ± 2	3.52 ± 0.12
Composite-3	10.97 ± 1.74	0.36 ± 0.07	47 ± 2	1.87 ± 0.19
Pt thin film	16.49 ± 1.63	0.53 ± 0.09	63 ± 2	5.53 ± 0.24

Note: The data uncertainty arises from conducting two separate experiments on the DSSC device under similar conditions.

The photoconversion efficiency (*η*), quantification of the amount of incident photon converted into electrical energy, is explicitly defined as.

$\text{Efficiency},\eta =\frac{P_{\max}}{P_{\text{input}}}$, where $P_{\max}=V_{\text{OC}}\times I_{\text{SC}}\times FF$

(2) $$\text{or},\eta =\frac{\;V_{\text{OC}}\times I_{\text{SC}}\times FF}{P_{\text{input}}}$$

For this study, input power ($P_{\text{input}})$ is 100 mWcm^−2^ i.e., 1 kWm^−2^.

Table 2 clearly indicates that Composite-1 exhibits the highest PCE (4.25 ± 0.32 %) which is close to the Pt thin film (5.53 ± 0.24). These efficiency results align with the conclusions drawn from all other electrochemical analyses, including CV, EIS, and Tafel polarization plots. Consequently, composite-1 demonstrates superior performance, achieving a PCE of 4.25 ± 0.32 %, followed by composite-2 with 3.52 ± 0.12 %, and composite-3 with the lowest performance at 1.87 ± 0.19 % PCE. The PCE of the f-MWCNT/Nr-GO-21 ternary composite is 3.91 ± 0.20 %, surpassing that of composite-2 and composite-3, yet falling short of composite-1. This suggests that the incorporation of a small amount of TiO_2_ enhances the structural integrity of the composite without causing a significant reduction in its surface area. The performance of this quaternary composite CE based DSSC is comparable to the Pt thin film-based system (*η* = 5.53 %) (see supporting information for more details, Fig. S14). The obtained performance of this quaternary composite is comparable to the other composite materials reported in the literature (Table S1).

The superior PCE of composite-1 is attributed to its higher surface area, structural integrity, and higher chemical stability compared to the other composites. The cyclability of the cell employing composite-1 as the CE was evaluated through 500 reversible redox cycles (Fig. S15). Remarkably, no notable changes were observed in the post 500 redox cycles, and the system retained its capability to generate electricity, evidenced by the persistence of four redox processes in the cyclic voltammogram (Fig. S15). In addition, the material collected from the electrode surface after 500 cycles was subjected to chemical analysis, specifically FTIR spectroscopy. No discernible changes in the properties of the composite material were detected in the FTIR spectrum (Fig. S16), indicating its stability and robustness over 500 repeated cycles of operation.

## 4 Conclusions

This study introduces a novel f-MWCNT/Nr-GO/TiO_2_ quaternary composite counter electrode for DSSCs. The electrochemical investigation has extensively examined the integration of these distinct components and their impact on the electrocatalytic process. EIS studies confirm that the addition of 25 % PEDOT:PSS effectively enhances the functionalization of MWCNT, resulting in improved composite formation and a high level of MWCNT dispersion, while maintaining its electrical conductivity. Electrochemical analysis shows that adding a small amount of TiO_2_ to the f-MWCNT/Nr-GO composite boosts cell efficiency to 9 %. Electron delocalization in the TiO_2_ nanostructure increases the electrocatalytic reaction rate, thereby improving cell efficiency. Moreover, the electron-donating N atom of Nr-GO, crystalline indices of TiO_2_, and conducting polymer boost carrier density and mobility on the composite surface. The rapid charge mobility identified through EIS analysis accelerates $I_{3}^{-}$ reduction, enhancing cell efficiency. In summary, these distinctive features of the quaternary composite make it an outstanding CE, offering durability over 500 cycles, long-lasting performance, and high photoconversion efficiency. The efficiency of composite-1 (4.25 ± 0.32 %) is like that of the Pt thin film CE (5.53 ± 0.24 %). We propose that further advancements in the cell engineering of this composite material could result in increased efficiency. This study highlights the role of composite materials in advancing DSSCs and their potential to innovate renewable energy technologies for sustainable solutions.

## CRediT authorship contribution statement

**A M Mahmudul Hasan:** Writing – review & editing, Writing – original draft, Methodology, Investigation, Formal analysis, Data curation. **Md. Abu Bin Hasan Susan:** Writing – review & editing, Validation, Supervision, Resources, Project administration, Funding acquisition, Conceptualization.

## Data availability

Experimental data from this study are available from the corresponding author upon request.

## Declaration of competing interest

The authors declare that they have no known competing financial interests or personal relationships that could have appeared to influence the work reported in this paper.
