# Supplementary material for: PEDOT:PSS polymer functionalized carbon nanotubes integrated with graphene oxide and titanium dioxide counter electrode for dye-sensitized solar cells
Source: Heliyon. 2025 Jan 25;11(3):e42272. doi: 10.1016/j.heliyon.2025.e42272 (PMC11808681; doi:10.1016/j.heliyon.2025.e42272)
Supplement: Multimedia component 1 [file mmc1.docx]

**Electronic Supplementary Information**

**PEDOT:PSS Polymer Functionalized Carbon Nanotubes Integrated with Graphene Oxide and Titanium Dioxide Counter Electrode for Dye-Sensitized Solar Cells**

A M Mahmudul Hasan^1^, Md. Abu Bin Hasan Susan^1,2*^

*^1^Department of Chemistry, Faculty of Science, University of Dhaka, Bangladesh*

*^2^Dhaka University Nanotechnology Centre, University of Dhaka, Bangladesh*

**Email: susan@du.ac.bd*

**Characterization of Materials**

Fourier transform infrared (FTIR) spectra of the materials were obtained using a Perkin Elmer FTIR spectrophotometer (Model: Shimadzu, Japan). Samples were prepared as KBr pellets with a ratio of 100:1 KBr to sample. Spectra were recorded in the wavenumber range of 4000-450 cm^-1^. The hydrodynamic sizes of the titanium dioxide nanorods (TiO_2_ NR) were measured using a Zetasizer Nano ZS90 (ZEN3690, Malvern Instruments Ltd, UK). The measurements were conducted with a He-Ne laser beam of wavelength 632.8 nm and a fixed scattering angle of 90°. Morphological analysis of the synthesized TiO_2_ NR, nitrogen-doped reduced graphene oxide, and composites was performed using a JEOL Analytical Scanning Electron Microscope (Model JSM-6940LA). The acceleration voltage of the electron gun was set to 20 kV with a probe current of 1.0 nA. Magnifications ranging from 10,000 to 100,000 were utilized, and images were captured without sputtering, with samples mounted on conducting carbon tape. Energy-Dispersive X-ray Spectroscopy (EDS) was also aligned with the SEM instrument. Thermogravimetric analysis of the synthesized materials was conducted using a Thermal Analyzer (TG-DTA 7200, Hitachi, Japan). The experiment was carried out from ambient temperature to 900 °C in a ceramic pan at a heating rate of 10 °C/min under an N_2_ atmosphere. Surface area and pore size distribution of the materials were determined using a N_2_ sorption BET surface area analyzer (Belsorp mini-2, BEL, Japan). UV spectra were recorded using a Spectro UV-visible double beam spectrophotometer (Model: UVD-500).


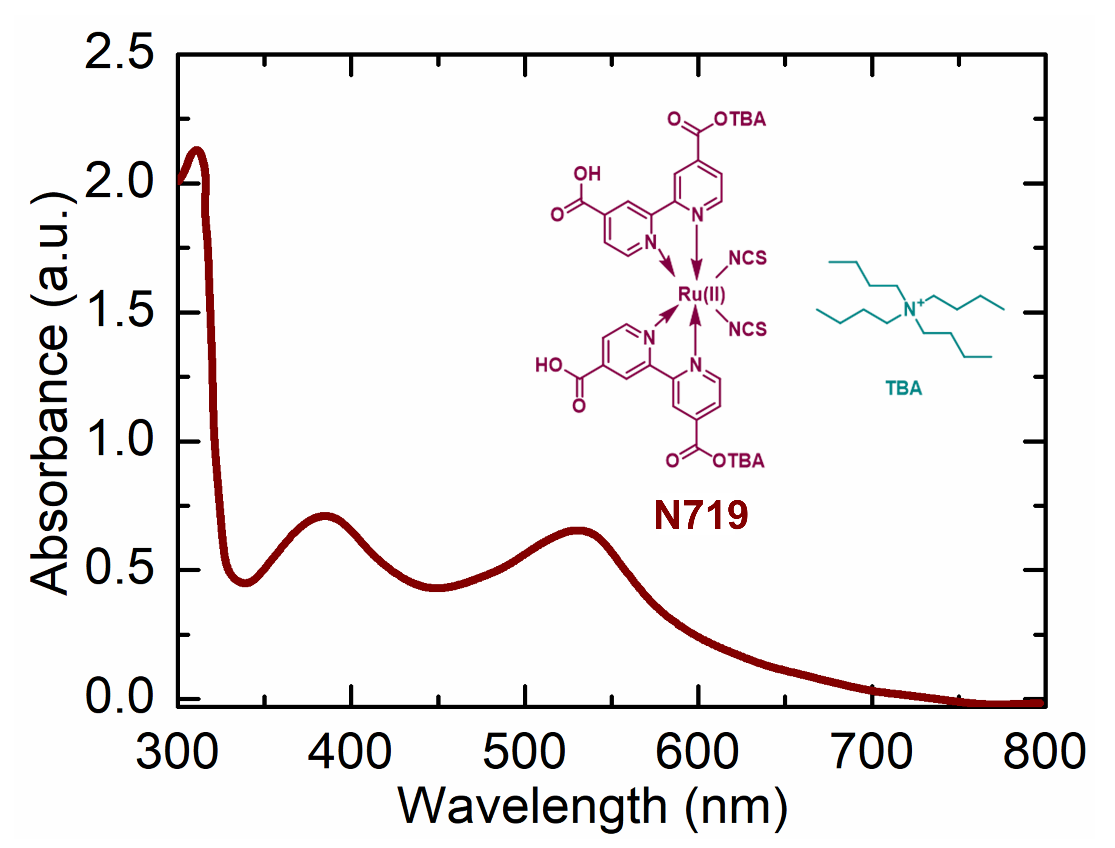


**Figure S1**. UV-Visible spectrum of N719 dye solution.





**Figure S2:** FTIR spectra of commercial TiO_2_ and TiO_2_ nanorods.





**Figure S3.** DLS profiles of TiO_2_ calcined at different temperatures.


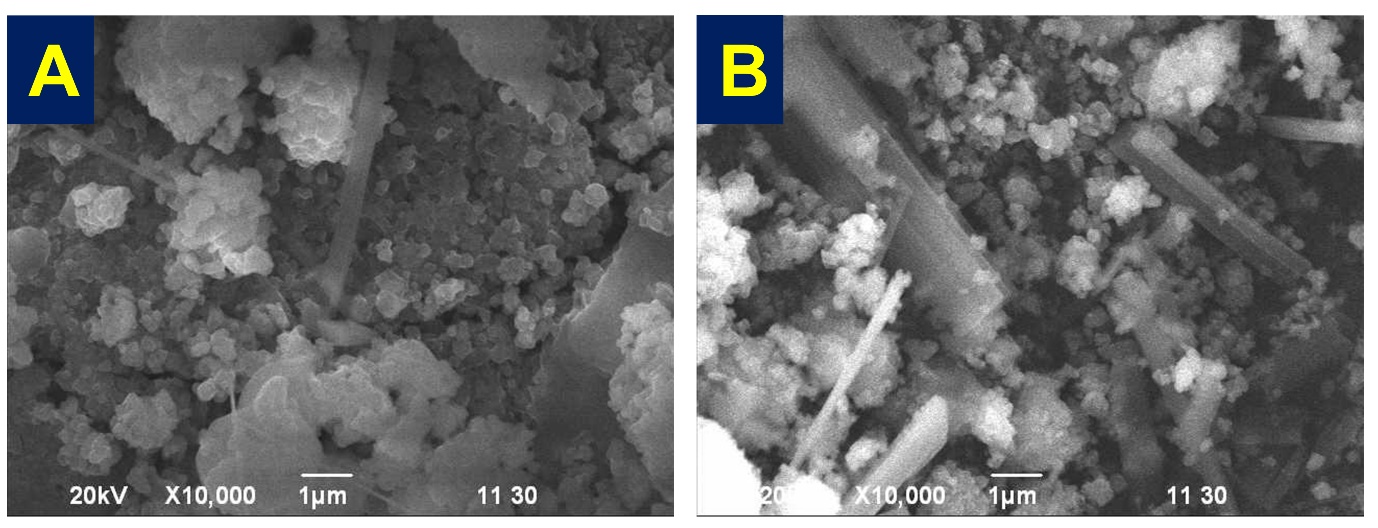


**Figure S4.** SEM images of (A) TiO_2_ NRs calcined at 400 °C and (b) TiO_2_ NR calcined at 600 °C.


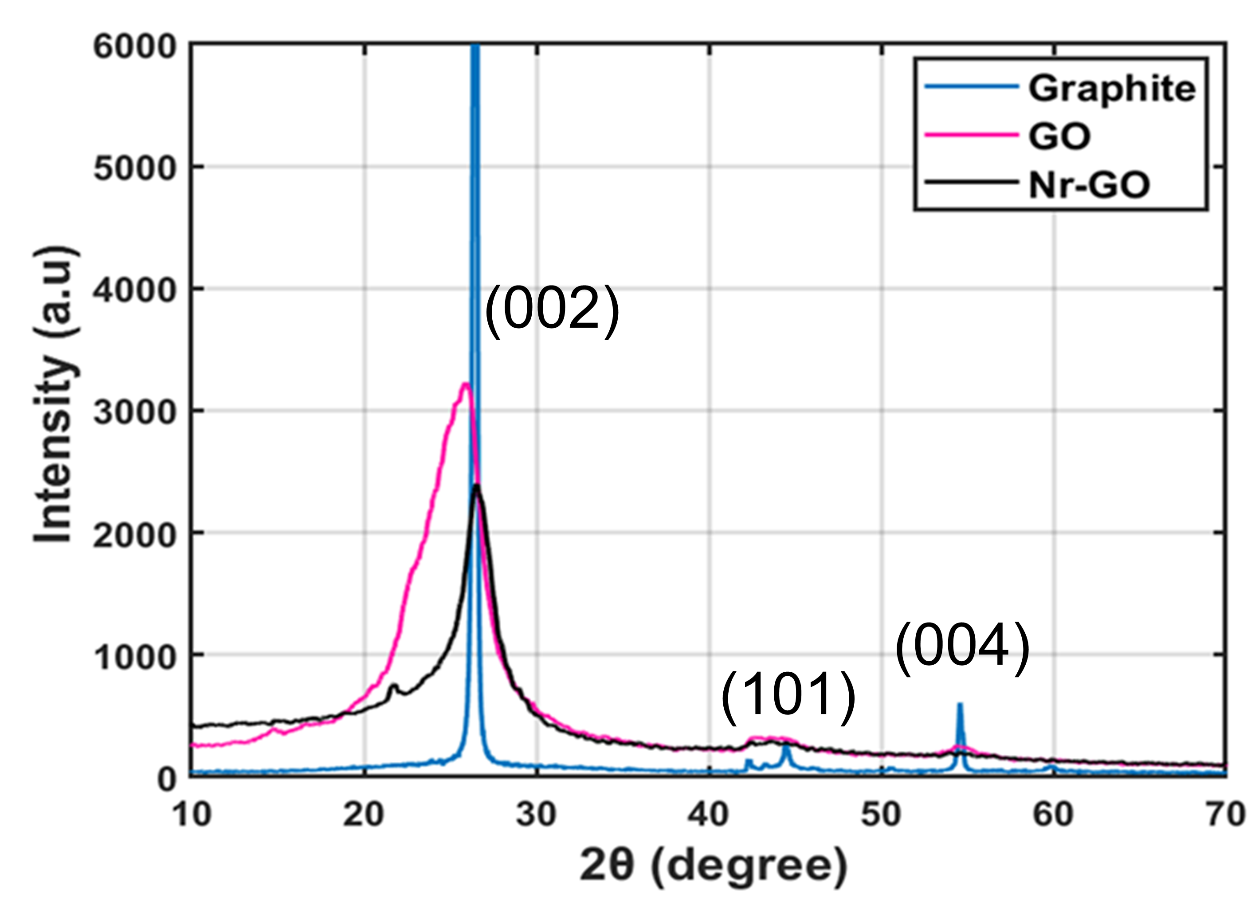


**Figure S5.** X-ray diffraction patterns of graphite, GO, and Nr-GO.





**Figure S6.** Barrett–Joyner–Halenda (BJH) plots showing the pore size distribution of MWCNT, MWCNT-PSSNa, and Nr-GO.





**Figure S7.** Barrett–Joyner–Halenda (BJH) plots showing the pore size distribution of synthesized composites.


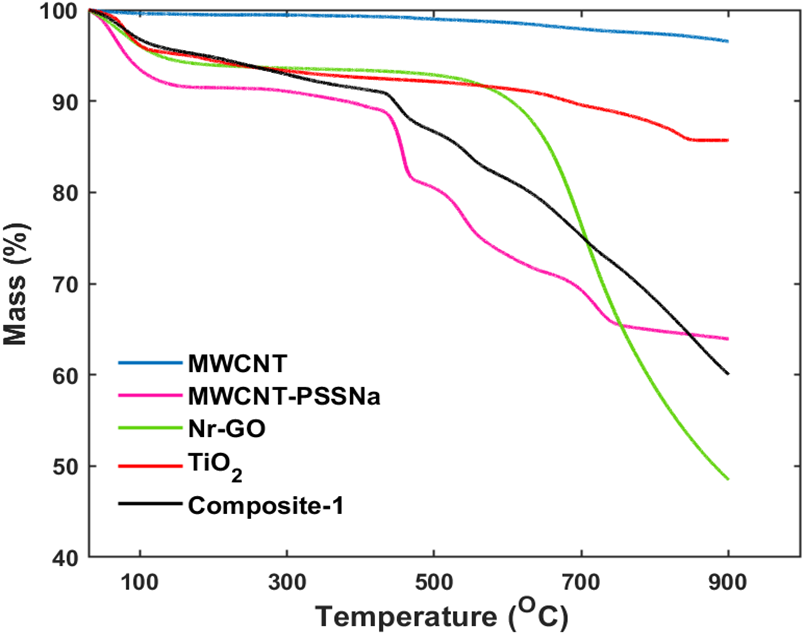


**Figure S8.** Thermograms of MWCNT, MWCNT-PSSNa, Nr-GO, TiO_2_ NR, and f-MWCNT/Nr-GO/TiO_2_ composite at the ambient atmospheric condition.

**

**

**Figure S9.** Background CV of 0.5 M LiI on the bare Pt disk working electrode at 50 mV s^-1^.

**
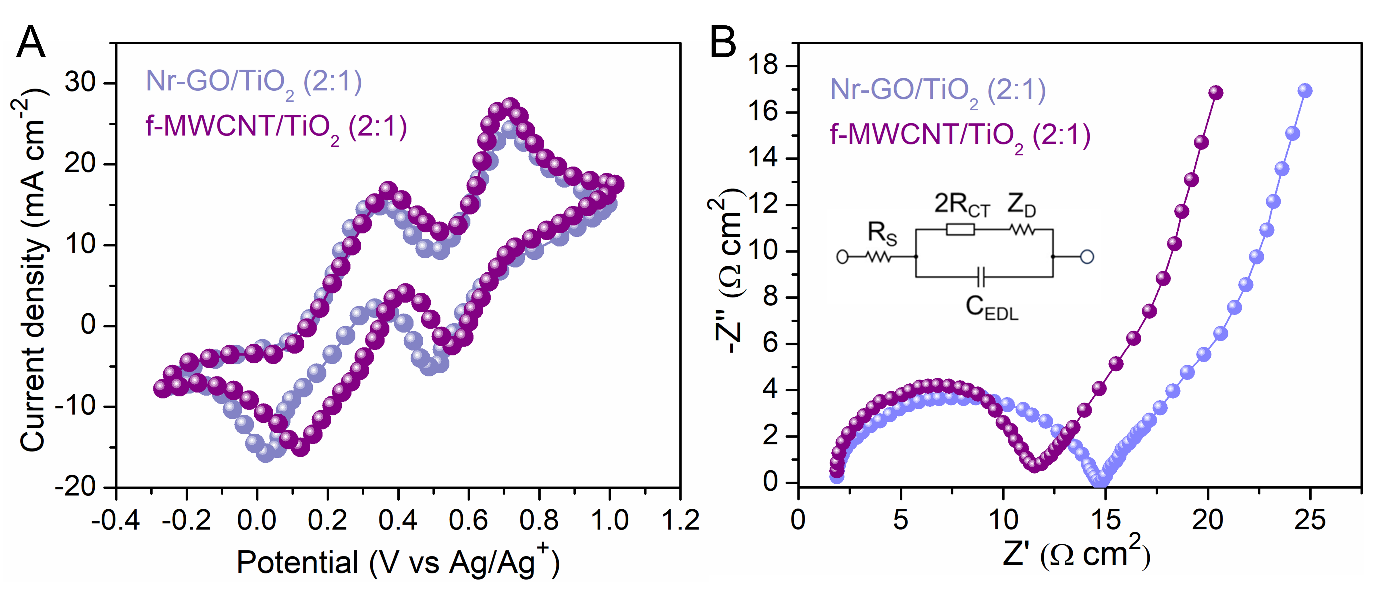
**

**Figure S10. (A)** Cyclic voltammogram and (B) Nyquist plot of Nr-GO/TiO_2_ (2:1) and f-MWCNT/TiO_2_ (2:1) binary composites in 0.5 M LiI, 0.05 M I_2_, and 0.1 M LiClO_4_ in acetonitrile electrolyte solution. (CV and EIS were recorded at 50 mV s^-1^ scan rate and 1 to 10^6^ Hz frequency respectively).

**Active surface area determination of the composite electrode**

The active surface area of the three quaternary composites in the electrochemical system were determined by the Rundles Sevick equation: *i*_p_=(2.69×105)n^3/2^ AD^1/2^ ν^1/2^ C

Where *i*_p_ is the peak current, ν^1/2^ is the scan rate, n is the number of electron transfers in a redox event, C is the solution concentration and D is the ion diffusion coefficient and A is the active electrode surface area. We used an identical 0.5 M LiI, 0.05 M I_2_, and 0.1 M LiClO_4_ in acetonitrile electrolyte solution, so the concentration and the ion diffusion coefficient are constant for all three composite systems. So, the steeper slope for a specific composite material will denote the comparatively higher active electrochemical surface.

After plotting the CV profiles at different scan rates, the fitted data shows a maximum slope for composite-1 and lowest for composite-3 indicating their corresponding bigger and smaller electrochemical surface area.

**
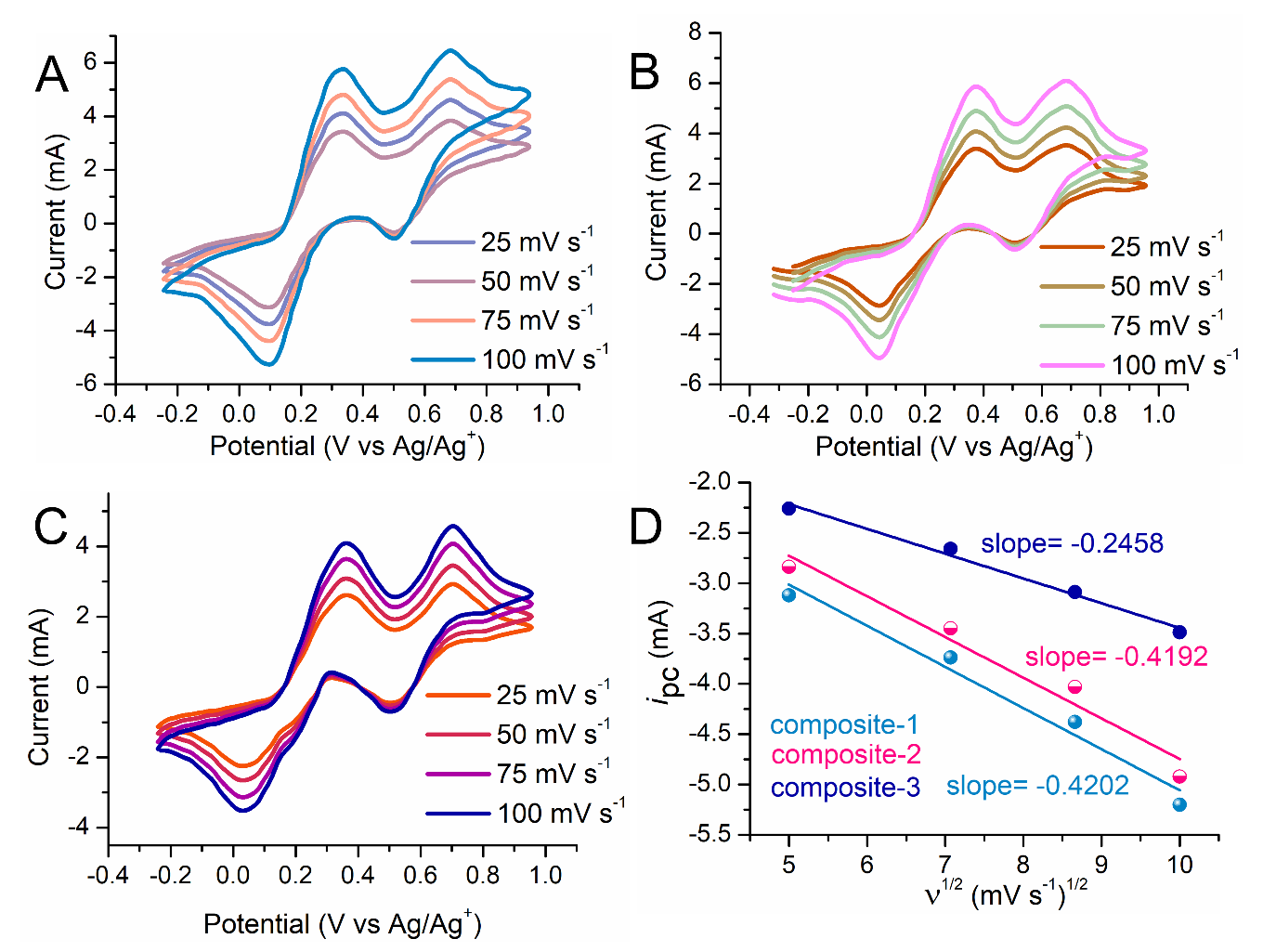
**

**Figure S11.** Cyclic voltammograms of (A) quaternary composite-1 (B) composite-2 and (C) composite-3 recorded at different scan rates in 0.5 M LiI, 0.05 M I_2_, and 0.1 M LiClO_4_ in acetonitrile electrolyte solution. (D) i_pc_ vs ν^1/2^ plot for the 2^nd^ reduction of the three composites obtained from the CVs.

**Electrode Preparation and DSSC Assembly.**

| **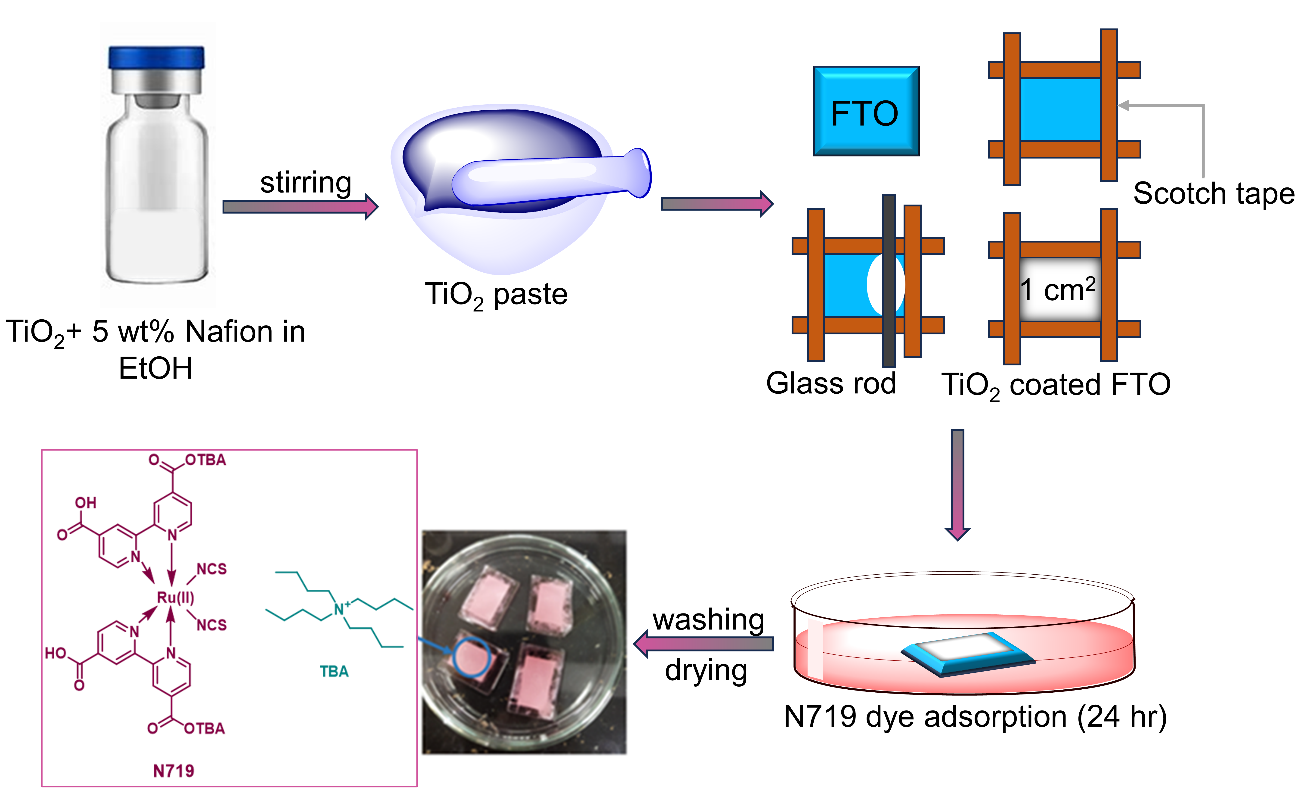** |
| --- |

**Figure S12.** Dye sensitized TiO_2_ photoanode preparation for DSSC.

**
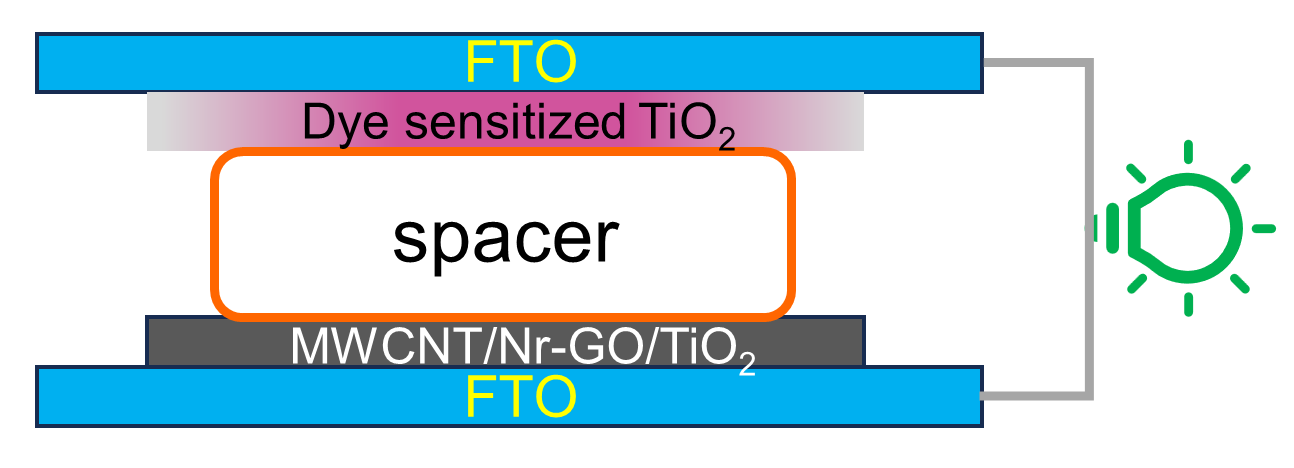
**

**Figure S13.** A schematic diagram of DSSC assembly used in this study.

**Pt thin film preparation and performance Analysis**

The Pt catalyst was deposited on the FTO using the thermal decomposition process. The FTO glass was heated to a temperature of 470 °C for a duration of 15 minutes after being cleaned. A 0.9 mM solution of H_2_PtCl_6_. 6H_2_O in ethanol was applied to the FTO substrates by drop casting. The substrates were then heated to 470 °C and annealed for 15 minutes.





**Figure S14.** *J-V* curve characteristics of Pt thin film counter electrode based DSSC.





**Figure S15.** Stability test of the material by 500 CV cycles of the DSSC with composite-1 counter electrode.





**Figure S16.** FTIR spectra of composite-1 before and after 500 CV cycling tests.

| **Table S1.** Photovoltaic properties of dye-sensitized solar cells constructed using various composite counter electrodes. | | | | | |
| --- | --- | --- | --- | --- | --- |
| CE Materials | J_SC_ (mA cm^-2^) | V_OC_ (mV) | FF (%) | PCE (η) % | Ref |
| Pt/TiO2/WO2 | 12.54 | 830 | 70 | 7.23 | S1 |
| Pt/NiO/Ag | 30.10 | 810 | 46 | 11.27 | S2 |
| TiN/PEDOT:PSS | 14.45 | 727 | 67 | 7.06 | S3 |
| PEDOT:PSS/graphene | 15.70 | 770 | 65 | 7.31 | S4 |
| RGO/SWCNT | 12.81 | 860 | 76 | 8.37 | S5 |
| r-GO/MnO₂/NiO/CuO | 13.46 | 750 | 76 | 7.67 | S6 |
| Ni–PANI–G | 12.92 | 772 | 53 | 5.30 | S7 |
| CoS/PEDOT:PSS | 12.53 | 773 | 69 | 6.31 | S8 |
| Carbon/TiO_2_ | 12.53 | 700 | 57 | 5.50 | S9 |
| SiO_2_/PEDOT:PSS | 13.50 | 720 | 58 | 5.66 | S10 |
| f-MWCNT/Nr-GO/TiO_2_ | 14.88 | 560 | 53 | 4.25 | This work |

**Supporting References:**

1. Y. Wang, C. Zhao, M. Wu, W. Liu, T. Ma, Highly efficient and low cost Pt-based binary and ternary composite catalysts as counter electrode for dye-sensitized solar cells, Electrochimica Acta, 105 (2013) 671-676.
2. Z. Lan, L. Que, W. Wu, J. Wu, High-performance Pt-NiO nanosheet-based counter electrodes for dye-sensitized solar cells, Journal of Solid State Electrochemistry, 20 (2016) 759-766.
3. H. Xu, X. Zhang, C. Zhang, Z. Liu, X. Zhou, S. Pang, X. Chen, S. Dong, Z. Zhang, L. Zhang, P. Han, X. Wang, G. Cui, Nanostructured Titanium Nitride/PEDOT:PSS Composite Films As Counter Electrodes of Dye-Sensitized Solar Cells, ACS Applied Materials & Interfaces, 4 (2012) 1087-1092.
4. G. Yue, J. Wu, Y. Xiao, J. Lin, M. Huang, Z. Lan, L. Fan, Functionalized graphene/poly(3,4-ethylenedioxythiophene):polystyrenesulfonate as counter electrode catalyst for dye-sensitized solar cells, Energy, 54 (2013) 315-321.
5. H. Zheng, C.Y. Neo, J. Ouyang, Highly Efficient Iodide/Triiodide Dye-Sensitized Solar Cells with Gel-Coated Reduce Graphene Oxide/Single-Walled Carbon Nanotube Composites as the Counter Electrode Exhibiting an Open-Circuit Voltage of 0.90 V, ACS Applied Materials & Interfaces, 5 (2013) 6657-6664.
6. P. Joselene Suzan Jennifer, S. Muthupandi, M. Joe Raja Ruban, S. Prathap, J. Madhavan, M. Victor Antony Raj, A quaternary nanocomposite as an efficient counter electrode for Pt-free Dye-sensitized solar cells (DSSC), Materials Letters, 340 (2023) 134151.
7. X. Chen, J. Liu, K. Qian, J. Wang, Ternary composites of Ni–polyaniline–graphene as counter electrodes for dye-sensitized solar cells, RSC Advances, 8 (2018) 10948-10953.
8. G. Yue, J. Wu, Y. Xiao, J. Lin, M. Huang, Z. Lan, Application of Poly(3,4-ethylenedioxythiophene):Polystyrenesulfonate/Polypyrrole Counter Electrode for Dye-Sensitized Solar Cells, The Journal of Physical Chemistry C, 116 (2012) 18057-18063.
9. P. Joshi, Y. Xie, M. Ropp, D. Galipeau, S. Bailey, Q. Qiao, Dye-sensitized solar cells based on low cost nanoscale carbon/TiO2 composite counter electrode, Energy & Environmental Science, 2 (2009) 426-429.
10. D. Song, M. Li, Y. Li, X. Zhao, B. Jiang, Y. Jiang, Highly Transparent and Efficient Counter Electrode Using SiO2/PEDOT–PSS Composite for Bifacial Dye-Sensitized Solar Cells, ACS Applied Materials & Interfaces, 6 (2014) 7126-7132.
